# Thiamine Supplementation Alleviates Lipopolysaccharide-Triggered Adaptive Inflammatory Response and Modulates Energy State via Suppression of NFκB/p38 MAPK/AMPK Signaling in Rumen Epithelial Cells of Goats

**DOI:** 10.3390/antiox11102048

**Published:** 2022-10-18

**Authors:** Yi Ma, Mawda Elmhadi, Chao Wang, Zelin Li, Hao Zhang, Banglin He, Xiujuan Zhao, Zhenbin Zhang, Hongrong Wang

**Affiliations:** 1 Laboratory of Metabolic Manipulation of Herbivorous Animal Nutrition, College of Animal Science and Technology, Yangzhou University, Yangzhou 225009, China; 2 School of Biomedical Sciences, The University of Western Australia, M Block, Queen Elizabeth II Medical Centre, Nedlands, WA 6009, Australia; 3 Faculty of Veterinary and Agricultural Sciences, The University of Melbourne, Shepparton, VIC 3647, Australia

**Keywords:** thiamine, rumen epithelial cells, inflammation, energy metabolism, mitochondrial function

## Abstract

Studies have shown that exogenous thiamine (THI) supplementation can alleviate inflammation and promote rumen epithelial development in goats and cows. This research aimed to evaluate the effect of THI supplementation on LPS-induced inflammation and energy metabolic dysregulation in RECs of goats. Cells were stimulated with either 5 μg/mL THI for 18 h (THI group) or with 5 μg/mL LPS for 6 h (LPS group). The CON group was stimulated with DMEM/F-12 medium without THI for 18 h. The LPTH group was pretreated with THI for 18 h, followed by LPS stimulation for 6 h. THI supplementation decreased the ROS content (*p* < 0.05), as well as the ratios of phosphorylated (p)-p65 to p65 (*p* < 0.05) and p-AMPKα to AMPKα (*p* < 0.05). Interestingly, when the p38 gene was overexpressed in the LPTH group, the ratio of p-p65 to p65 and p-AMPKα to AMPKα proteins significantly increased, and ATP content decreased (*p* < 0.05). Our results suggest that THI possesses anti-inflammatory and metabolic-modulatory effects in RECs. The mechanism is largely related to the suppression of the NF-κB/p38 MAPK/AMPK signaling pathway. Additionally, we also revealed that THI supplementation can inhibit LPS-induced oxidative damage and apoptosis to protect mitochondrial function in RECs.

## 1. Introduction

Acute or subacute ruminal acidosis (ARA or SARA) has long been defined as a digestive disorder that affects ruminants [1]. The dietary transition from forage to high concentrate (HC) diets, comprising abundant rapidly fermentable carbohydrates or even excessive intake of these carbohydrates over a long period, changes the ruminal fermentation pattern towards non-fibrous carbohydrate (NFC) fermentation [2]. These carbohydrates can be quickly fermented and converted into short-chain fatty acids and lactate, which accumulate in the rumen, resulting in lower ruminal pH than in the normal physiological environment (pH < 5.6) [3]. A direct implication of feeding high concentrate diets is an enormous risk of rumen and systemic metabolic disorders. In addition, SARA is related to inefficient feed efficiency and major financial losses, likely explained by decreased fermentation efficiency in the rumen, but also by systemic inflammation and interrelated systemic effects of SARA [4]. Research has shown that the sudden acid load in the rumen may damage the epithelial barrier function [4]. Moreover, an increased rumen concentration of microbe-associated molecular pattern (MAMP) molecules, such as lipopolysaccharides (LPSes) and histamine, has been demonstrated in ruminants fed HC diets, potentially due to increased Gram-negative bacterial lysis [5].

The ruminal epithelium, as a natural barrier, plays a crucial role in preventing the invasion of LPS into the body’s circulatory system. Unfortunately, following damage of the rumen epithelial barrier function, LPS may be translocated into the interior circulation of the body which leads to changes in the levels of acute-phase proteins and the occurrence of a systemic inflammation response [6]. LPS binds to specific pattern recognition receptors (PRRs), such as toll-like receptor 4 (TLR4), and interacts with rumen epithelial cells (RECs), thereby triggering an immune response [7]. This impairs epithelial protection, resulting in inflammation, oxidative stress (OS), energy metabolism disturbance, and mitochondrial damage [8,9,10,11]. Furthermore, studies have revealed that LPS intensifies nuclear factor kappa-B (NF-κB) expression, which enhances inflammatory response [12]. The transcription of NF-κB can also be promoted by the activation of mitogen-activated protein kinase 14 (MAPK14, alternatively known as p38) [13]. In addition, NF-κB can be activated by oxidative stress resulting in cytotoxicity and recruitment of apoptotic factors [14]. As the primary organelle involved in the regulation of oxidative damage (OD), mitochondria can produce both reactive oxygen species (ROS) and adenosine triphosphate (ATP)—therefore, the function of mitochondria are closely connected with the use of nutrients to produce ATP and dissipate ROS as a byproduct [15]. However, pro-inflammatory NF-κB signaling causes mitochondrial dysfunction in response to cellular overloading.

The physiological function of thiamine (THI; also known as vitamin B1) in energy metabolism via participation in the pentose phosphate pathway (PPP) and tricarboxylic acid (TCA) cycle has been extensively researched [10]. It is easily absorbed from the digestive tract and converted into thiamine pyrophosphate (TPP), which plays a crucial role as a cofactor for enzymes of energy metabolic pathways involved in the generation of ATP [16]. Numerous studies have revealed that THI deficiency can reduce ATP production and lead to an imbalance in energy metabolism [17,18]. AMP-activated protein kinase (AMPK) can be activated when low ATP levels inhibit cellular energy consumption to regulate cellular responses to the metabolic disturbance [19,20]. THI is also used to increase antioxidant formation and NADPH levels by the PPP pathway [21]. In addition, numerous studies have mentioned that exogenous THI supplementation can alleviate inflammation induced by LPS and promotes rumen epithelial development in goats and cows [9,10,22,23]. However, studies have shown that feeding an HC diet for a long period can trigger an increase in the degradation of thiamine by thiaminase, as well as lysis and death of thiamine, producing bacteria in the rumen [9,24], which may cause THI deficiency.

Based on this evidence, we hypothesized that THI supplementation could alleviate inflammation and modulate energy metabolism by suppressing the NF-κB/p38 MAPK/AMPK signaling pathway. According to multiple studies on THI in goats conducted by our research group [9,10,23], rumen epithelial cells (RECs) of goats were used as trial objects in current study. The objective of this study was to investigate the association between THI treatment, LPS-induced inflammatory response, and energy metabolism disturbance.

## 2. Materials and Methods

### 2.1. Reagents and Chemicals

Dulbecco’s modified Eagle medium (DMEM)/Nutrient Mixture F-12 (F12) and fetal bovine serum (FBS) were purchased from Gibco (Carlsbad, CA, USA). Antibiotics, THI (T4625, purity ≥ 99%), L-glutamine, HEPES, insulin-transferrin-selenium (ITS), epidermal growth factor (EGF), and 2.5% trypsin solution were obtained from Sigma-Aldrich (St. Louis, MO, USA).

### 2.2. Cell Culture

Primary RECs were separated from the rumen epithelial tissue of Boer goats using a repeated 2.5% trypsin digestion protocol according to a previous study [25]. The isolated RECs were cultured in T25 cell culture flask (Corning Inc., Corning, NY, USA) with 5 mL DMEM/F12, 10% FBS, 1.36 mM L-glutamine, 20 mM HEPES, antibiotics (100 μg/mL streptomycin, 0.25 μg/mL amphotericin B, 240 U/mL nystatin, 50 mg/L gentamycin, 100 mg/L kanamycin), 1% ITS solution, and 10 ng/mL EGF. The cell medium was replaced every 2 to 3 d. The RECs were purified using a series of 2.5% trypsin enzymatic digestions to eliminate fibroblast contamination. Before each digestion experiment, the cell pellet was washed and centrifuged three times with sterile phosphate buffer solution (PBS) containing Ca^2+^ and Mg^2+^. After 2 weeks of culture, the absence of other hybrid cells was verified using a light microscope. The immunofluorescence experiment with cytokeratin 18 protein was performed to verify that cells were indeed RECs (Figure S1). In addition, the ideal metabolic functionality of the RECs was also evaluated based on the methodology of Gao (2020) (data not shown) [26]. The RECs were incubated with a growth medium for passaging, collected at the third generation, and stored in liquid nitrogen with mixed cell cryopreservation fluid (a solution containing 10% dimethyl sulfoxide, 50% FBS, and 40% DMEM/F12) via a gradient freezing protocol. Cryopreserved cells were resuscitated at 37 °C and then passaged to the fifth generation for each subsequent experiment.

### 2.3. Experimental Design

**Figure 1:**
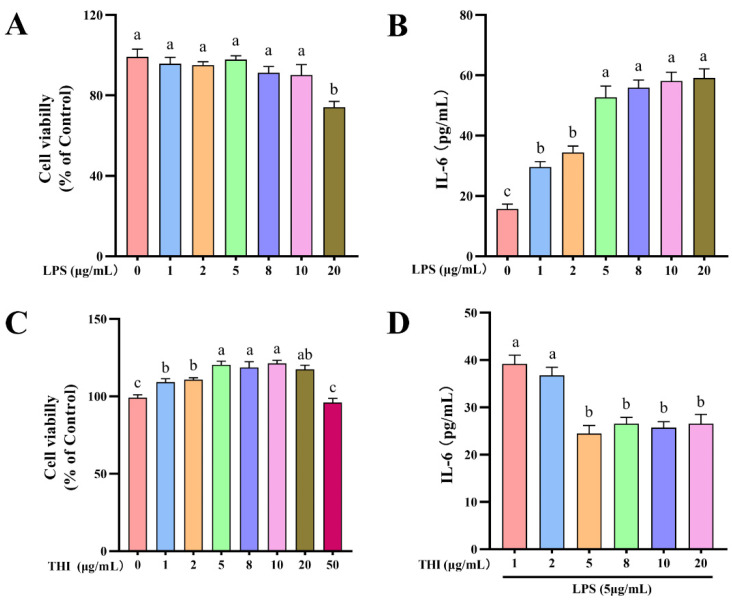
Cell viability and IL-6 secretion of cells treated with different concentrations of lipopolysaccharide (LPS) and thiamine (THI). (**A**) Cell viability of cells treated with different concentrations of LPS. (**B**) IL-6 secretion in culture media after different concentrations of LPS. (**C**) Cell viability of cells treated with different concentrations of THI. (**D**) IL-6 secretion in culture media after different concentrations of THI and 5 μg/mL LPS. Cell was treated with 0 μg/mL THI or 0 μg/mL LPS as a control, and cell viability is expressed as a percentage of control. Data are presented as means ± SEM (standard error of the mean), *n* = 3/group (results representative of at least three independent experiments); CON group, no added LPS or THI; THI group, 5 μg/mL THI; LPS group, 5 μg/mL LPS; LPTH group, 5 μg/mL THI and 5 μg/mL LPS. Each column with different letters (a–c) are significantly different (*p* < 0.05).

Custom-formulated THI-deficient DMEM/F12 media (BasalMedia Technologies, Shanghai, China) supplemented with 10% FBS contains ~10 nM THI (data not shown). However, this was ineffective for reaching complete THI-deficient conditions. Therefore, THI deficiency in the culture medium was achieved using 10 μM pyri-THI hydrobromide (PT) and THI pyrophosphate kinase-1 inhibitor (Sigma Aldrich, St. Louis, MO, USA), which inhibits the conversion of THI to its activated state, THI pyrophosphate [27]. The LPS used in each experiment was obtained from Escherichia coli O55:B5 lyophilized powder (L6529; Sigma-Aldrich, St. Louis, MO, USA). Cellular inflammatory models were established and optimized with respect to LPS and THI concentrations, and the timing of inflammatory responses. The cells were stimulated with either THI for 18 h (THI group), LPS for 6 h (LPS group), or with DMEM/F-12 medium without THI for 18 h (CON group). A final group was pretreated with THI for 18 h, followed by washing and then LPS stimulation for 6 h (LPTH group). The cells were treated with optimized doses of 5 μg/mL LPS and/or 5 μg/mL THI based on the data of a dose-dependence experiment (Figure 1). Before treatment, the cells were starved for 6 h to minimize the total amount of THI within the cells.

### 2.4. Cell Viability

RECs were seeded into 96-well plates (Corning Inc., Corning, NY, USA) with an optimized density (1 × 104 cells/mL) and grown with different concentrations of LPS and THI for 6 and 18 h, respectively (Figure 1). According to the manufacturer’s protocols, cell viability was determined using a Cell Counting Kit-8 (Vazyme Biotech, Nanjing, China). The absorbance of all samples was read at 450 nm using a microplate reader (Gen 5; BioTek Instruments, Winooski, VT, USA). Cell viability was analyzed as a percentage relative to the control cells unexposed to THI or LPS.

### 2.5. ELISA for IL-6 Concentrations

According to the manufacturer’s instructions, the ELISA experiment was conducted to determine IL-6 concentrations in the culture medium using ELISA IL-6 kits (Abcam, Cambridge, UK). Briefly, samples were incubated with standards for 2 h, followed by the addition of detection antibodies and incubated for 1 h at room temperature. At the end of the incubation, chromogenic agent was added to the sample and incubated for 20 min in the dark. The absorbance at 450 nm was measured using a microplate reader (Gen 5; BioTek Instruments, Winooski, VT, USA).

### 2.6. Apoptotic Cell Number Analysis by Flow Cytometry and Fluorescence Microscopy

The apoptotic effect on RECs was measured using an Annexin V-FITC/PI Apoptosis Detection Kit (A211; Vazyme Biotech, Nanjing, China). Cells were collected using 2.5% trypsin solution and stained by fluorescein isothiocyanate (FITC)/propidium iodide (PI). Next, all samples were analyzed for apoptosis by flow cytometry (FACSAria SORP; BD Biosciences, Franklin Lakes, NJ, USA) and fluorescence microscopy (DMi8; Leica, Wetzlar, Germany). FlowJo v10.7.1 (BD Biosciences, Franklin Lakes, NJ, USA) was used to evaluate the apoptosis rate of cells.

### 2.7. Determination of ROS Content by Flow Cytometry and Fluorescence Microscopy

The ROS content in cells was evaluated using the ROS assay kit (CA1410; Solarbio, Beijing, China), following the manufacturer’s instructions. Each sample was analyzed for ROS content by flow cytometry (FACSAria SORP; BD Biosciences, Franklin Lakes, NJ, USA) and fluorescence microscopy (DMi8; Leica, Wetzlar, Germany). FlowJo v10.7.1 (BD Biosciences, Franklin Lakes, NJ, USA) was used to evaluate the fluorescence intensity of ROS in cells.

### 2.8. Mitochondrial Membrane Potential (MMP, ΔΨm) by Flow Cytometry and Fluorescence Microscopy

The ΔΨm of RECs was detected using an MMP assay kit (CA1310, Solarbio, Beijing, China) following the manufacturer’s instructions. Briefly, cells were collected using 2.5% trypsin solution and incubated at 37 °C for 20 min with 500 μL 5,5′,6,6′-tetrachloro-1,1′,3,3′-tetraethyl-imidacarbocyanine (JC-1). The cells were then measured for ΔΨ m by flow cytometry (FACSAria SORP; BD Bioscience) and fluorescence microscopy (DMi8; Leica). FlowJo v10.7.1 (BD Biosciences, Franklin Lakes, NJ, USA) was used to analyze ΔΨm in cells.

### 2.9. Immunofluorescence Staining

RECs (1 × 10^6^ cells/well) were seeded into 6-well plates (Corning Inc., Corning, NY, USA), fixed with 4% paraformaldehyde (Solarbio) for 20 min, and then permeabilized with 0.5% Triton X-100 (Solarbio) for 15 min at 37 °C. The cells were sealed with 5% BSA at 37 °C for 30 min, then incubated at 4 °C overnight with primary antibody (p-IκB, 1:100, AF1870; Beyotime, Shanghai, China; p-AMPKα, 1:100, AF2677; Beyotime, Shanghai, China). Afterward, the cells were washed three times with PBS (Solarbio), then incubated in the dark at 37 °C for 1 h with secondary antibody (1:100; S0006 and S0018; Affinity Biosciences, Cincinnati, OH, USA). Then, 100 nM DAPI (Beyotime, Shanghai, China) was used for nuclear counterstaining for 3 min. Finally, the cells were observed and imaged under a fluorescence microscope (DMi8; Leica, Wetzlar, Germany).

### 2.10. Analysis of Mitochondrial Morphology by Transmission Electron Microscope (TEM)

The mitochondrial morphology of RECs was determined based on a prior research method [28]. In brief, cells were fixed with ice-cold 2% glutaraldehyde, then dehydrated sequentially using acetone in different concentrations (30 min steps), and impregnated with epoxy resin using 25, 50, and 75% acetone-resin (1 h duration) steps to 100% resin for 3 h at 60 °C. Finally, sections were cut, stained, and imaged using a TEM (Tecnai G230; FEI NanoPorts, Hillsboro, OR, USA) with an accelerating voltage of 80 kV.

### 2.11. Mitochondrial DNA (mtDNA) Content Measurement

Total DNA in cells was extracted using the QIAamp DNA Mini Kit (Vazyme Biotech, Nanjing, China), and the mtDNA copy number was determined by real-time PCR (RT-PCR), with genomic DNA being used as the loading control. The sequences of primers were as follows: mitochondria DNA loop, 5′-ACAAACTTCCCACTCCACAAGCC-3′ (forward), 5′-GTGTAGGCGAGCGGTGTAATGTAC-3′ (reverse). (Gene accession number: GenBank: AB162217.1) The relative copy number of mtDNA was quantified by the ratio of mtDNA to nuclear DNA [29].

### 2.12. Measurement of Oxidative Stress Markers

According to previous research, the levels of malonaldehyde (MDA), lactate dehydrogenase (LDH), catalase (CAT), superoxide dismutase (SOD), glutathione peroxidase (GSH-Px), and total antioxidant capacity (T-AOC) were determined using a commercial kit (Nanjing Jiangcheng Biotechnology Institute, Nanjing, China) [30].

### 2.13. ATP Determination

The ATP content was quantified using a commercial ATP determination kit (Solarbio) according to the manufacturer’s instructions and following a prior study [31]. All values were shown as fold-changes relative to the CON group.

### 2.14. RT-PCR Analysis on Gene Expression

Total RNA was extracted from the treated RECs by FastPure Cell/Tissue Total RNA Isolation Kit (RC101; Vazyme Biotech, Nanjing, China) following the manufacturer’s instructions. The RNA integrity was measured by NanoDrop 2000 (Thermo Fisher Scientific, Waltham, MA, USA) and gel electrophoresis. RT-PCR was conducted as previously described [9,10]. The designer primers (Sangon Biotech, Shanghai, China) for housekeeping genes and target genes are shown in Supplementary Table S1. Amplification efficiency for the primer was evaluated using a serial dilution of pooled cDNA samples. Based on Gao (2013), the first-rank housekeeping genes (ACTB, GAPDH, and HPRT) were evaluated by determining the candidate genes ranking, and the order of gene expression stability [32]. The genes from most to least stable were HPRT1, GAPDH, and ACTB. Thus, ACTB was chosen as the reference gene to normalize mRNA expression. The RT-PCR data were normalized using the 2^−ΔΔCt^ method compared with the ACTB genes [33].

### 2.15. Western Blot Analysis

Western blot analysis was performed as described previously [9]. The antibody information used in the experiment is shown below: p38 (#8690S, 1:1000; Cell Signaling Technology, Danvers, MA, USA), Bcl-2 (AF6139, 1:1000; Affinity Bioscience), Bax (#2772, 1:1000; Cell Signaling Technology), AMPKα (#5823, 1:1000; Cell Signaling Technology), p-AMPKα (#11818, 1:1000; Cell Signaling Technology), SIRT1 (#2493, 1:1000; Cell Signaling Technology), PGC1α (AF5395, 1:1000; Affinity), TLR4 (AF7017, 1:1000; Affinity), p65 (#8242, 1:1000; Cell Signaling Technology), p-p65 (#3033, 1:1000; Cell Signaling Technology), p-IKBα (#2859, 1:1000; Cell Signaling Technology), β-actin (AC038, 1:1000; ABclonal, Woburn, MA, USA), and horseradish peroxidase (A0208, 1:1000; Beyotime, Shanghai, China). Differences in protein transfer efficiency between blots were normalized based on the quantification of β-actin.

### 2.16. Cell Transfection

The total length of the p38 gene in goats is 3866 bp (XM_018038791.1), with the CDS area ranging between 475–1557 bp. Primers were designed and synthesized according to the CDS region, and the DNA in the target fragments was amplified and purified. Subsequently, the target gene fragment was cloned into the pcDNA 3.1 vector and sequenced by COME. RECs were transfected with plasmid DNA using GP-transfect-Mate (GenePharma, Shanghai, China) following the manufacturer’s protocols. Cells were treated with either LPS or THI based on the above details after transfection, and used in subsequent analyses [34].

### 2.17. Statistical Analysis

The data are shown as the mean and pooled standard error of the means (SEM). Cell viability, IL-6 concentration, and analysis after p38 gene overexpression were calculated using one-way ANOVA with Duncan’s multiple range test by SPSS v21.0 (IBM, Armonk, NY, USA) to evaluate the difference between different treatment groups (labeled means without a common letter are significantly different, *p* < 0.05). Other studies were subjected to two-way ANOVA analysis, and the statistical model included the effects of LPS, THI, and their interactions. For significant interactions, a post hoc test was conducted using Bonferroni’s multiple comparison test (* *p* < 0.05). Statistical significance was set at *p* < 0.05. The experiments were conducted in triplicate, and all experiments were repeated at least three times.

## 3. Results

### 3.1. REC Viability with LPS and THI Treatments at Different Doses

The CCK-8 experiment was performed to determine cell viability after treatment with LPS and THI for 6 and 18 h, respectively. Concentrations of LPS at 0, 1, 2, 5, 8, and 10 μg/mL made no significant difference regarding cell viability (*p* > 0.05, Figure 1A). THI concentrations showed variable effects on cell viability, with 5, 8, 10, and 20 μg/mL having remarkably higher cell viability (*p* < 0.05) in comparison to concentrations of 0, 1, 2, and 50 μg/mL (Figure 1C). In addition, ELISA analysis showed IL-6 levels in the culture medium were significantly higher (*p* < 0.05) at LPS concentrations of 5, 8, 10, and 20 μg/mL than at 0, 1, or 2 μg/mL (Figure 1B). In contrast, THI supplementation (5, 8, 10, and 20 μg/mL) significantly decreased IL-6 concentration in 5 μg/mL LPS-treated cells (*p* < 0.05, Figure 1D). These results reveal that the doses of 5 μg/mL LPS and 5 μg/mL THI did not have a cytotoxic effect on the RECs, and the inflammation model meets the purpose of the stud.

### 3.2. THI Inhibits Apoptosis Induced by LPS in RECs

**Figure 2:**
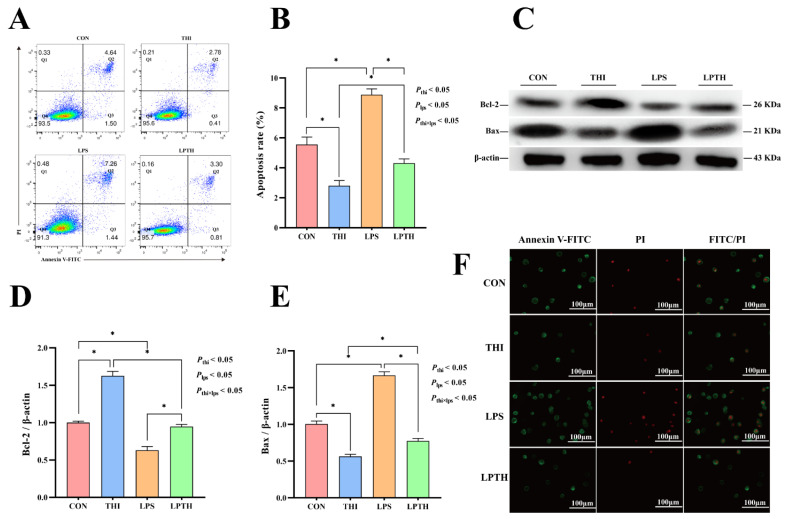
Thiamine (THI) inhibits apoptosis induced by lipopolysaccharide (LPS) in RECs. (**A**) Fluorescent staining with fluorescein isothiocyanate (FITC)/propidium iodide (PI). Green stain: apoptosis; green and red stain: necrosis. (**B**) Flow cytometry scatter plots with FITC/PI staining. Q1, necrosis; Q2, late apoptosis; Q3, live cells; Q4, early apoptosis. (**C**) The apoptosis percentages in cultures with different treatments. (**D**) Representative Western blots for Bcl−2 and Bax protein levels with different treatments. (**E**,**F**) Immunoblotting and measurement of the intensity. Protein levels were normalized to the relative abundance of β−actin. Data are presented as means ± SEM (standard error of the mean), *n* = 3/group (results representative of at least three independent experiments); CON group, no added LPS or THI; THI group, 5 μg/mL THI; LPS group, 5 μg/mL LPS; LPTH group, 5 μg/mL THI and 5 μg/mL LPS. * denotes *p* < 0.05, significant difference.

To determine the effect of LPS (5 μg/mL) and THI (5 μg/mL) on REC apoptosis, we used flow cytometry and fluorescence detection experiments. THI deficiency resulted in a significant apoptotic rate compared with the THI group (*p* < 0.05, Figure 2A–C). LPS stimulation induced a more significant apoptotic rate on RECs than in the CON group (*p* < 0.05). In addition, the level of Bax protein was significantly increased and Bcl-2 decreased in the LPS group compared with the CON group (*p* < 0.05; Figure 2D–F). Interestingly, THI co-supplementation (LPTH) reversed LPS-induced apoptosis and changed the level of these proteins compared with the LPS group (*p* < 0.05).

### 3.3. THI Inhibits OD Induced by LPS in RECs

**Figure 3:**
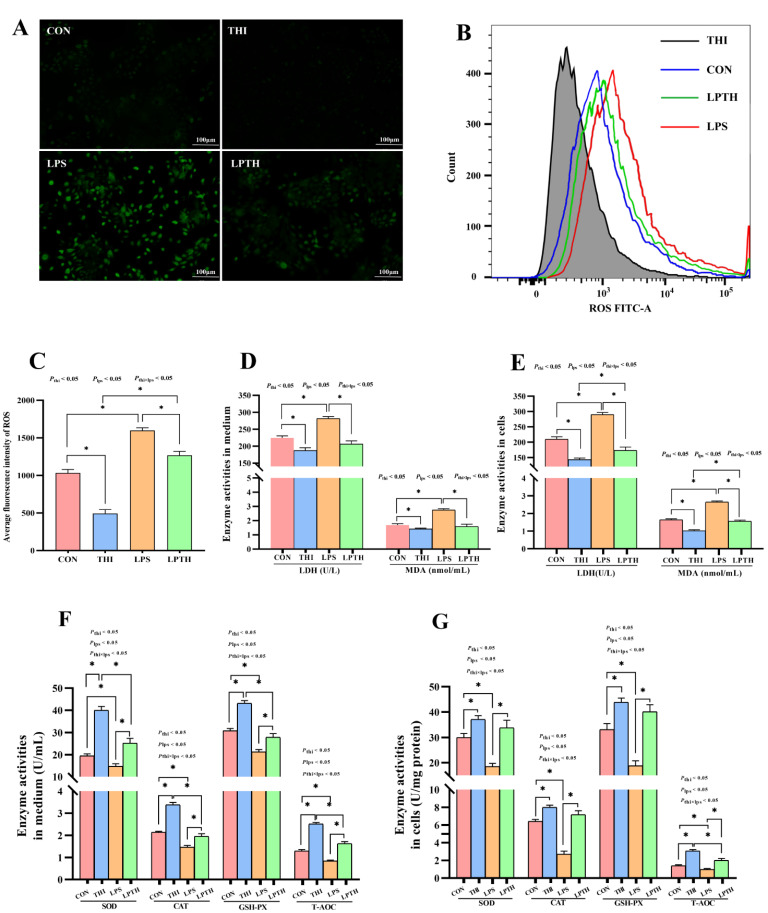
Thiamine (THI) inhibits oxidative damage induced by lipopolysaccharide (LPS) in RECs. (**A**) Fluorescent staining of reactive oxygen species (ROS) with DCFH-DA. Green staining: ROS. (**B**) Flow cytometry plots of ROS with DCFH-DA staining. (**C**) Flow cytometry merge plots of ROS for different treatments. (**D**) Average fluorescence intensity of ROS. (**E**,**F**) Enzyme activities of LDH and MDA in medium and cells. ((**G**), Enzyme activities of SOD, CAT, GSH-PX, and T-AOC in medium and cells. MDA, malonaldehyde; LDH, lactate dehydrogenase; SOD, superoxide dismutase; CAT, concentrations catalase; GSH-PX, glutathione peroxidase; T-AOC, total antioxidant capacity. Data are presented as means ± SEM (standard error of the mean), *n* = 3/group (results representative of at least three independent experiments); CON group, no added LPS or THI; THI group, 5 μg/mL THI; LPS group, 5 μg/mL LPS; LPTH group, 5 μg/mL THI and 5 μg/mL LPS. * denotes *p* < 0.05, significant difference.

To verify the effect of THI on the cellular OD of RECs (essentially proliferation prevention), the ROS content and oxidation-related enzyme activities were detected. As expected (Figure 3), compared to the CON group, THI treatment markedly decreased ROS content in the mitochondria, LDH and MDA enzyme activities (*p* < 0.05) in the medium and cells, and increased SOD, CAT, GSH-PX, and T-AOC enzyme activities (*p* < 0.05) in the medium and cells. In contrast, stimulation of LPS significantly increased the ROS content, LDH, and MDA enzyme activities (*p* < 0.05) in the medium and cells, and significantly decreased SOD, CAT, GSH-PX, and T-AOC enzyme activities (*p* < 0.05) in the medium and cells compared to the CON group. Interestingly, the LPTH group treatment noticeably decreased ROS content in the mitochondria, and LDH and MDA enzyme activities (*p* < 0.05) in the medium and cells, while increasing SOD, CAT, GSH-PX, and T-AOC enzyme activities (*p* < 0.05) in the medium and cells compared with the LPS group.

### 3.4. THI Supplementation Maintains Mitochondrial Function in RECs

**Figure 4:**
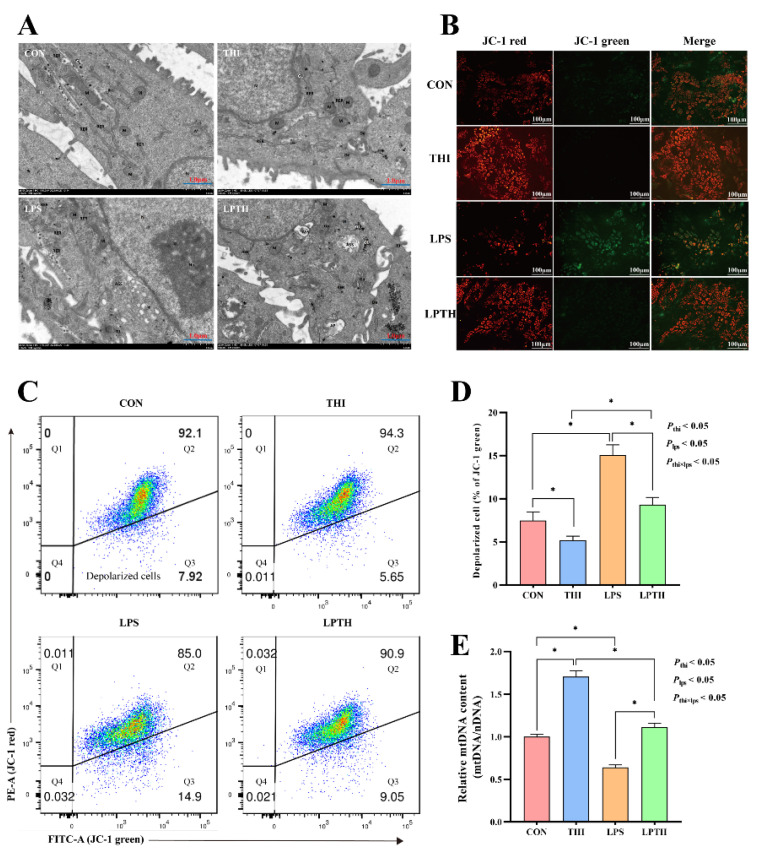
Thiamine (THI) supplementation maintains mitochondrial function in RECs. (**A**) Transmission electron microscope (TEM) images of cells with different treatments. TJ, tight junction; N, nucleus; M, mitochondria; RER, rough endoplasmic reticulum; GL, glycogenosome; Ass, autolysosome; De, desmosome; Go, Golgi apparatus; AP, autophagosome. (**B**) Fluorescent staining of mitochondrial membrane potential (MMP) with JC-1. JC-1 aggregates exhibited red fluorescence, and JC-1 monomers exhibited green fluorescence. (**C**) Flow cytometry scatter plots with JC-1 staining. Q3: depolarized cells. (**D**) The ratio of depolarized cells for different treatments. (**E**) Mitochondrial DNA content for different treatments. Data are presented as means ± SEM (standard error of the mean), *n* = 3/group (results representative of at least three independent experiments); CON group, no added LPS or THI; THI group, 5 μg/mL THI; LPS group, 5 μg/mL LPS; LPTH group, 5 μg/mL THI and 5 μg/mL LPS. * denotes *p* < 0.05, significant difference.

To evaluate the role of THI in cell states and define the functional status of mitochondria under stimulation of LPS, we measured the ultrastructure and MMP of RECs. TEM of REC cross-sections within the CON group showed integrity and normal nuclei, but a small number of mitochondria (Figure 4A). In the THI group, integrated cell membranes could be observed, along with abundant mitochondria, and more ribosomes in the rough endoplasmic reticulum. On the contrary, in the LPS group, the cells with mild edema showed an enlarged nucleus, few mitochondria, and a locally dilated rough endoplasmic reticulum. As expected, in the LPTH group the mitochondria were abundant and the rough endoplasmic reticulum normal, although the cells had slight edemas. Furthermore, compared to the CON group, the THI group had a dramatic decrease in the collapse of the MMP and an increased mtDNA content (*p* < 0.05) in RECs. In contrast, the LPS group showed a more significant increase in the collapse of the MMP and a decrease in the mtDNA content (*p* < 0.05) than the CON treatment. Interestingly, the LPTH group significantly preserved the MMP and increased the mtDNA content (*p* < 0.05) compared with the LPS group (Figure 4B–E).

### 3.5. THI Supplementation Alleviates the Adaptive Response to Inflammation Induced by LPS and Promotes the Expression of THI Transporters in RECs

**Figure 5:**
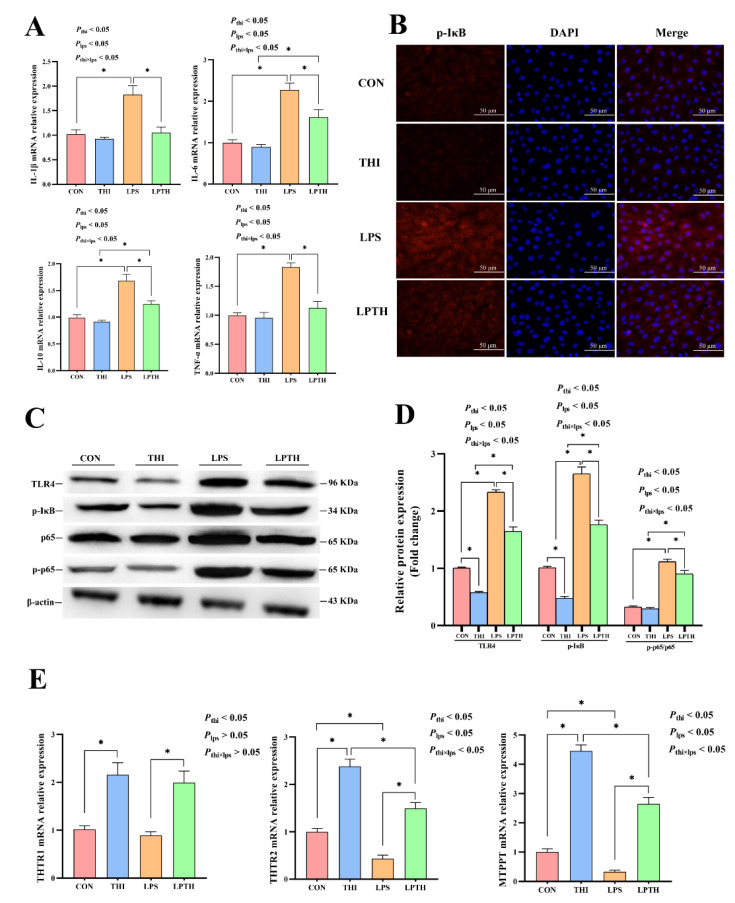
Thiamine (THI) supplementation alleviates inflammation induced by lipopolysaccharide (LPS) and promotes the expression of THI transporters in RECs. (**A**) The expression of IL-1β, IL-6, IL-10, and TNF-α genes with different treatments. (**B**) Immunofluorescence staining of p-IκB protein with different treatments. Red: IκB protein; Blue: nuclei. (**C**) Representative Western blots for TLR4, phosphorylated IκB (p-IκB), p65, and phosphorylated p65 (p-p65) protein with different treatments. (**D**) Immunoblotting and measurement of the intensity. Protein levels were normalized to the respective abundance of β-actin. (**E**) The expression of *THTR1*, *THTR2*, and *MTPPT* genes with different treatments. Data are presented as means ± SEM (standard error of the mean), *n* = 3/group (results representative of at least three independent experiments); CON group, no added LPS or THI; THI group, 5 μg/mL THI; LPS group, 5 μg/mL LPS; LPTH group, 5 μg/mL THI and 5 μg/mL LPS. * denotes *p* < 0.05, significant difference.

The gene expression of inflammatory factors is presented in Figure 5A. Compared to the CON group, THI treatment did not significantly affect the expression of inflammatory cytokines in the RECs (*p* > 0.05). As expected, the LPS stimulation induced an increase in *IL-1β*, *IL-6*, *IL-10*, and *TNF-α* gene expression (*p* < 0.05) and specifically triggered IκB and TLR4 signaling (*p* < 0.05) (Figure 5A,B). In contrast, compared with the LPS group, THI supplementation reversed the expression for the above-mentioned genes (*p* < 0.05). Next, the protein levels of TLR4, phosphorylated NF-κB (p65 subunit), NF-κB (p65 subunit), and phosphorylated IκBα (p-IκBα) were examined (Figure 5C,D). The THI group decreased TLR4 and p-IκBα protein levels compared to the CON group (*p* < 0.05). Moreover, the LPS group significantly increased the ratio of p-p65/t-p65 and the above protein levels (*p* < 0.05) compared with the CON group. THI treatment reversed the LPS-induced inflammatory response, resulting in decreased ratios of p-p65/t-p65, as well as p-IκB and TLR4 protein levels in the LPTH group compared with those in the LPS group (*p* < 0.05). Furthermore, the immunofluorescence results also demonstrated that the LPTH group reliably inhibited activation of p-IκB, as indicated by it being found at lower levels than in the LPS group (Figure 5B).

Additionally, the results also showed that the THI treatment markedly increased the expression of *THTR2* and *MTPPT* genes compared to that of the CON group (*p* < 0.05, Figure 5E). Similarly, the LPS group also had a more significant decrease in the expression of the above genes than that of the CON group (*p* < 0.05). Conversely, the LPTH group had significantly increased expression of *THTR2* and *MTPPT* genes compared with the LPS group (*p* < 0.05).

### 3.6. THI Supplementation Modulates Energy Metabolism Disturbance Induced by LPS in RECs

**Figure 6:**
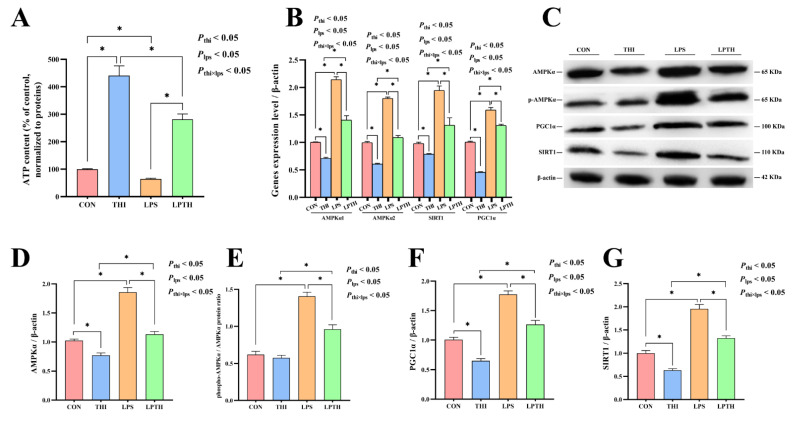
Thiamine (THI) supplementation modulates energy metabolism disturbance induced by lipopolysaccharide (LPS) in RECs. (**A**) ATP content for different treatments. (**B**) The expression of AMPKα1, AMPKα2, SIRT1, and PGC1α genes with different treatments. (**C**) Representative Western blots for AMPKα, phosphorylated AMPKα (p-AMPKα), SIRT1, and PGC1α protein levels with different treatments. (**D**–**G**) Immunoblotting and measurement of intensity. Protein levels were normalized to the respective abundance of β-actin. Data are presented as means ± SEM (standard error of the mean), *n* = 3/group (results representative of at least three independent experiments); CON group, no added LPS or THI; THI group, 5 μg/mL THI; LPS group, 5 μg/mL LPS; LPTH group, 5 μg/mL THI and 5 μg/mL LPS. * denotes *p* < 0.05, significant difference.

The effect of THI on REC energy metabolism was examined in the inflammatory model induced by LPS. The results showed that the THI group had an increase in ATP content (*p* < 0.05, Figure 6A) compared with the CON group, while LPS stimulation decreased ATP synthesis (*p* < 0.05). Conversely, the LPTH group showed an increase in ATP synthesis (*p* < 0.05) compared to the LPS group. RT-PCR data demonstrated that the gene expression of AMPKα1, AMPKα2, SIRT1, and PGC1α decreased in the THI group (*p* < 0.05) compared to the CON group (Figure 6B). Simultaneously, cells with LPS stimulation increased the gene expression of the above genes (*p* < 0.05) compared to that of the CON group, but THI co-supplementation reversed the gene expression changes of AMPKα1, AMPKα2, SIRT1, and PGC1α compared with the LPS group (*p* < 0.05).

Furthermore, we investigated the effect of THI on the protein levels of AMPKα, phosphorylated AMPKα, PGC1α, and SIRT1 by using Western blot analysis (Figure 6C–G). The protein levels of AMPKα, PGC1α, and SIRT1—which are related to energy metabolism—increased in the CON group (*p* < 0.05) compared to the THI group, while the LPS-induced group had significantly higher levels of the above proteins than in the CON group (*p* < 0.05). Meanwhile, the LPS-induced group also exhibited increased ratio of phosphorylated AMPKα to total AMPKα (*p* < 0.05). Surprisingly, compared to the LPS group, these protein levels decreased in the LPTH group (*p* < 0.05).

### 3.7. THI Supplementation Does Not Alleviate the Adaptive Response to Inflammation Induced by LPS in RECs under p38 Gene Overexpression

**Figure 7:**
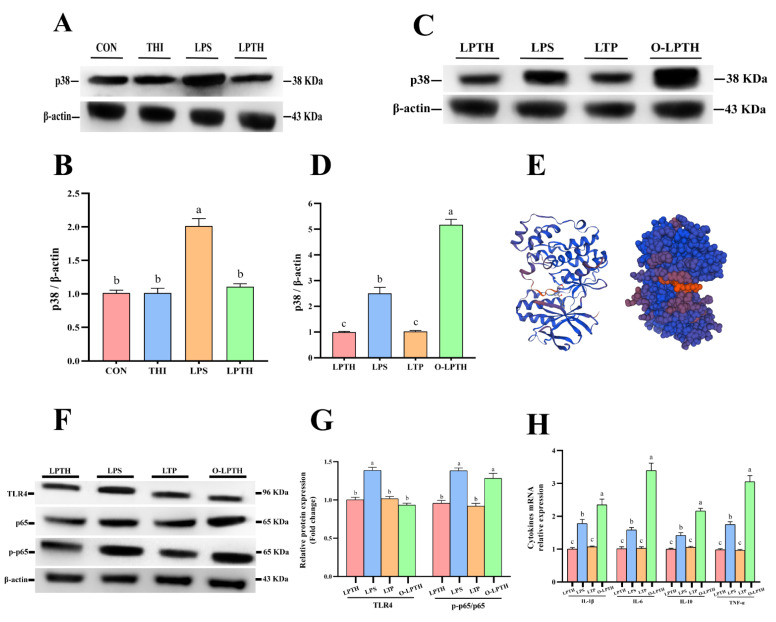
THI supplementation does not alleviate the adaptive response to inflammation induced by LPS in RECs under p38 gene overexpression. (**A**) Representative Western blots for p38 protein with different treatments. (**B**) Immunoblotting and measurement of the intensity for p38 protein. Protein expression was normalized by the respective abundance of β-actin. (**C**) Representative Western blots for p38 protein with different treatments in p38 gene overexpression RECs. (**D**) Immunoblotting and measurement of the intensity for p38 protein in p38 gene overexpression RECs. Protein expression was normalized by the respective abundance of β-actin. (**E**) p38 protein spatial structure model predicted by Swiss-model online tools (https://swissmodel.expasy.org). (**F**) Representative Western blots for TLR4, p65 and phosphorylated p65 (p-p65) protein with different treatments. (**G**) Immunoblotting and measurement of the intensity. Protein expression was normalized by the respective abundance of β-actin. (**H**) The expression levels of IL-1β, IL-6, IL-10 and TNF-α genes with different treatments. Data are presented as the mean ± SEM (standard error of the mean), *n* = 3/group; CON: Without LPS and thiamine; THI: contains 5 μg/mL thiamine without lipopolysaccharide; LPS: contains 5 μg/mL lipopolysaccharide without thiamine; LPTH: contains 5 μg/mL thiamine and 5 μg/mL lipopolysaccharide. LTP: cells were treated with 5 μg/mL thiamine and 5 μg/mL lipopolysaccharide after transfection with empty plasmid vector pcDNA 3.1. O-LPTH: cells were treated with 5 μg/mL thiamine and 5 μg/mL lipopolysaccharide after transfection with p38 gene. At least three independent experiments were performed. Each column with different letters (a–c) are significantly different (*p* < 0.05).

The levels of p38 protein are presented in Figure 7A,B. The LPS treatment increased the levels of p38 protein in RECs compared with the CON group (*p* > 0.05). As was expected, the LPTH group exhibited a decrease in p38 protein levels (*p* < 0.05). To further explore whether the inhibitory effect of THI on p38 causes these changes, we overexpressed the *p38* gene (Figure 7C,D). The O-LPTH group (cells treated with 5 μg/mL THI and 5 μg/mL LPS after transfection with the *p38* gene) increased the ratio of p-p65 to t-p65 compared to those in the LPTH group (*p* < 0.05, Figure 7F,G). Interestingly, TLR4 protein levels did not change significantly in the O-LPTH group compared to the LPTH group (*p* > 0.05). In addition, compared with the LPTH group, the O-LPTH group had a significantly increased gene expression of *IL-1β*, *IL-6*, *IL-10*, and *TNF-α* (*p* < 0.05, Figure 7H).

### 3.8. THI Supplementation Decreased Effects of Regulation in Energy Metabolism Disturbance Induced by LPS in RECs under p38 Gene Overexpression

**Figure 8:**
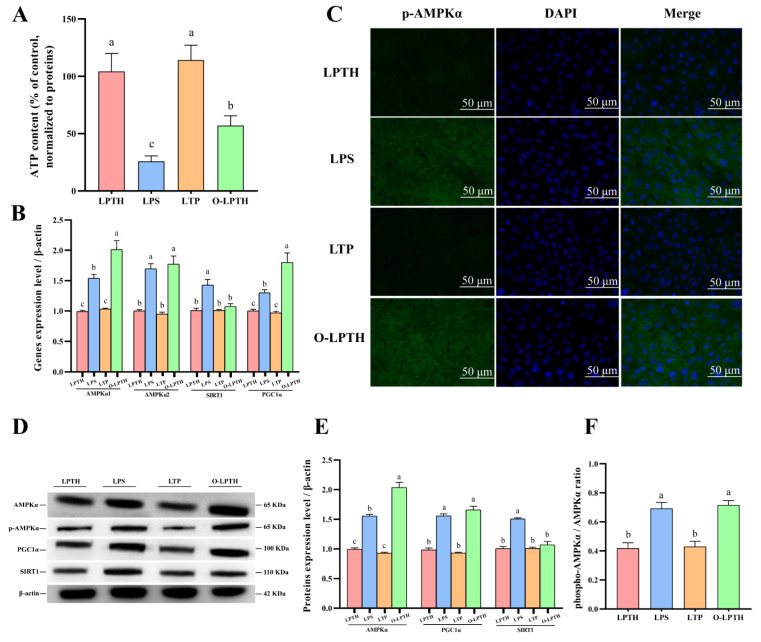
THI supplementation decreased effects of regulation in energy metabolism disturbance induced by LPS in RECs under p38 gene overexpression. (**A**) ATP content for different treatments. (**B**) Expression levels of AMPKα1, AMPKα2, SIRT1 and PGC1α genes with different treatments. (**C**) Immunofluorescence staining of p-AMPKα protein with different treatments. Green: p-AMPKα protein; Blue: nuclei. (**D**) Representative Western blots for AMPKα, phosphorylated AMPKα (p-AMPKα), SIRT1 and PGC1α protein with different treatments. (**E**,**F**) Immunoblotting and measurement of intensity. Protein expression was normalized by the respective abundance of β-actin. Data are presented as the mean ± SEM (standard error of the mean), *n* = 3/group; CON: Without LPS and thiamine; THI: contains 5 μg/mL thiamine without lipopolysaccharide; LPS: contains 5 μg/mL lipopolysaccharide without thiamine; LPTH: contains 5 μg/mL thiamine and 5 μg/mL lipopolysaccharide. LTP: cells were treated with 5 μg/mL thiamine and 5μg/mL lipopolysaccharide after transfection with empty plasmid vector pcDNA 3.1. O-LPTH: cells were treated with 5 μg/mL thiamine and 5 μg/mL lipopolysaccharide after transfection with p38 gene. At least three independent experiments were performed. Each column with different letters (a–c) are significantly different (*p* < 0.05).

The current results showed that the O-LPTH group underwent a decrease in ATP content (*p* < 0.05, Figure 8A) compared to the LPTH group. RT-PCR results demonstrated that the gene expression of *AMPKα1*, *AMPKα2*, and *PGC1α* increased (*p* < 0.05) for O-LPTH treatment compared to the LPTH group (Figure 8B). The protein levels of AMPKα and PGC1α and the ratio of phosphorylated AMPKα to total AMPKα were dramatically increased in the O-LPTH group compared to in the LPTH group (*p*< 0.05, Figure 8D–F). In addition, immunofluorescence results also demonstrated that the O-LPTH group had significantly increased activation of p-AMPKα protein in RECs compared to the LPTH group (Figure 8C).

## 4. Discussion

It is widely accepted that HC diets may lead to a range of metabolic dysfunction diseases in ruminants [35]. Specifically, the diet-induced low pH in the rumen damages the ruminal microbial community structure and increases the release of LPS [5]. The accumulated LPS and acid then synergistically injure the rumen barrier function [36]. Once the epithelium cells have been disrupted, local inflammation is activated by triggering the LPS/TLR4 signaling pathway. Thereafter, inflammatory cytokines are excessively released into the circulation system [12]. Meanwhile, overfeeding non-fibrous carbohydrates can also cause THI deficiency [9,37], which may be related to a decrease in THI production by bacteria such as Bacteroidetes, Fibrobacter, and Pyramidobacter [38]. Moreover, Zhu et al. (2015) demonstrated that LPS and inflammatory cytokines (IL-1β, IL-6, IL-10, and TNF-α) inhibit the gene expressions of THI transporters and receptors (such as THTR2) [39]. In the current study, RECs stimulated by LPS with THI supplementation had a higher gene expression of THTR2 and MTPPT, indicating that THI absorption was enhanced by THI supplementation.

THI supplementation has been shown to inhibit apoptosis and promote the proliferation of RECs [9,10]. In response to stimulation by LPS, inflammatory cytokines are expressed in RECs [40], which specifically bind receptors to activate the cell death pathway [41]. Furthermore, the Bcl-2 family factors including Bax and Bcl-2, as well as NF-κB are crucial regulators in the early stages of apoptosis [42,43]. In the present study, the Bax protein levels were significantly increased, whereas the Bcl-2 protein levels were significantly decreased in LPS-stimulated RECs. Meanwhile, THI deficiency also led to more REC apoptosis (CON treatment). Interestingly, these changes in apoptosis-related protein levels were significantly reversed in LPS-induced RECs treated with THI. These results further confirm the attenuating effect of THI on REC apoptosis. Apoptosis may be directly induced by mitochondrial damage, while ROS is an important factor causing mitochondrial dysfunction [44].

Under normal conditions, cells maintain a stable relationship between the production and neutralization of ROS by antioxidant pathways. Oxidative stress can occur in cells once ROS formation is accelerated by stimulation of LPS or when the free radical scavenging mechanisms are compromised [45]. ROS triggers apoptosis based on stimulation of plasma membrane death receptors. The activation of NF-κB induced by LPS elicits a systemic inflammatory process to release pro-inflammatory mediators, which result in excessive production of ROS and thus cause oxidative stress [46]. MDA, produced by ROS-mediated lipid peroxidation, is often used as a marker of OD. We measured the effect of THI on LPS-induced oxidative stress in RECs. The accumulation of ROS and MDA were confirmed in response to LPS and THI deficiency, along with a toxic reaction of LPS on RECs confirmed by a strong increase in LDH. In contrast, these negative effects could be reversed upon THI co-treatment. To realize whether the antioxidant function of THI is connected to ROS scavenging, we also examined the activities of antioxidant enzymes in cell environments in the medium and cells. As expected, the results demonstrated that the activities of CAT, T-AOC, SOD, and GSH-PX were significantly decreased in RECs with LPS stimulation, while the activities of these antioxidant enzymes were dramatically increased in RECs with THI co-treatment. It is worth mentioning that THI deficiency also reduced the activities of the above enzymes more so than in the THI group, which is consistent with our previous in vivo experiments on goats [10].

Mitochondria are the major source of ROS and the primary target for ROS-induced damage, leading to greater OD [47]. On the one hand, the release of ROS can decrease the MMP, which indicates mitochondrial impairment [48]. On the other hand, mitochondria play a major role in cellular energy production by producing ATP for various physiological pathways. In this regard, OD has also been confirmed to affect the generation of ATP in mitochondria [49]. Generally, the MMP level sharply declines with lower ATP content, thus indicating deterioration of mitochondrial function. Our results suggested that LPS-stimulated cells co-treated with LPS had higher MMP levels and ATP content than those with only LPS treatment. The TEM images also showed that THI supplementation could protect mitochondrial integrity and function. Furthermore, elevated ROS concentration in conjunction with reduced MMP levels results in mtDNA damage. According to the present results, the LPTH group had a higher mtDNA level than the LPS group. This further proves that THI plays a critical role in the maintenance of mitochondrial function.

AMPK plays an important role in mammalian cell adaptation to nutritional deprivation and pathological changes [50]. AMPK can be activated when low ATP levels inhibit cellular energy consumption to regulate cellular responses to the metabolic disturbance—in other words, it is activated in response to limited available energy [19]. In addition, AMPK can also promote the transcription of SIRT1 in response to energy deficiency, and enhance PGC1α deacetylation and activation, which is a necessary cellular response to mitochondrial metabolic disturbance [51]. The PGC1α gene plays a crucial role in regulating mitochondrial biogenesis involved in adaptive inflammation and glucose/fatty acid/energy metabolism [52]. As described above, THI is a cofactor for several enzymes related to carbohydrate metabolism, which involves oxidative decarboxylation of pyruvic acid and α-ketoglutaric acid to produce ATP [53]. THI deficiency causes inhibition of multiple oxidative decarboxylation pathways in the TCA cycle, which results in a decrease in ATP generation—in turn, activating more AMPK. In addition, LPS stimulation exacerbates ATP deficiency and induces AMPK expression. In the present study, the gene expression of AMPKα2, AMPKα1, PGC1α, and SIRT1 and the level of AMPKα phosphorylation showed a significant increase with LPS treatment compared to the THI group. In contrast, THI supplementation with LPS stimulation downregulated the above genes and protein levels in RECs and reduced the phosphorylation level of AMPKα. Thus, THI supplementation inhibited the AMPK signal transduction pathway in response to LPS, which is consistent with the increased ATP level.

Intestinal inflammation is largely associated with energy metabolism disruption and activation of AMPK [54]. Zhang et al. (2020) reported that THI supplementation dramatically alleviates rumen inflammation by decreasing the contents of the pro-inflammatory cytokines IL-6, TNF-α, and IL-1β both in the rumen epithelium and in serum of goats [23]. Similarly, in cow rumen epithelium, THI supplementation could suppress the LPS-induced inflammatory response, primarily through decreasing the LPS-induced NF-κB component p65 binding to promoter regions and expression of TLR4 receptor protein, thus inhibiting the excessive generation of inflammatory cytokines. As a part of the NF-κB complex in the cytoplasm, IκBα mainly launches NF-κB activity through dissociation of the NF-κB complex and nuclear translocation of phosphorylated NF-κB p65 [55]. In the current study, we confirm that THI restrains the secretion of phosphorylated NF-κB p65 into the nucleus. Moreover, the suppression of NF-κB activity is possibly modulated by the decreased phosphorylation of IκBα. These data show that THI alleviates the LPS-induced adaptive inflammatory response via inhibition of NF-κB activation, which may also be associated with the change in AMPK expression.

The p38 MAPK appears to be a vital regulator of gene transcription by modulating chromatin modifiers and remodelers. The promoters of several genes included in the inflammatory response, such as IL-6 or IL-8, present a p38 MAPK-dependent enrichment of histone H3 phosphorylation on Ser10 in LPS-stimulated cells. This phosphorylation promotes the activation of the cryptic NF-κB-binding sites, which represent promoters for increased NF-κB recruitment [56]. The p38 MAPK pathway can positively modulate NF-κB factor activity through various mechanisms, including chromatin remodeling by Ser10 phosphorylation of histone H3 at NF-κB-dependent promoters, or through direct/indirect regulation via IKK or p65 [57]. Furthermore, a previous study demonstrated that the Wip1 phosphatase can also lead to dephosphorylation of p38α, thus interfering with the positive effect of the p38 MAPK pathway on NF-κB signaling [58]. Our study indicated that LPS augmented the levels of MAPK p38 protein, resulting in the activation of NF-κB p65, whereas THI supplementation could reduce the LPS-induced p38 MAPK transcription.

To further explore whether the inhibition of p38 by THI causes an anti-inflammatory effect, we overexpressed the p38 gene. The current results indicated that THI treatment could not ameliorate the LPS-induced inflammatory response through inhibition of NF-κB activation in the LPTH group with p38 overexpression (LBP38 group). This shows that THI supplementation suppressed p38 MAPK expression, and thus failed to mediate the translocation of phosphorylated p65 into the nucleus. Moreover, our study also proved that the gene expression and protein levels of AMPKα2, AMPKα1, and PGC1α, as well as the level of AMPKα phosphorylation, were significantly increased in the LBP38 group compared to the LPTH and LPS groups.

In summary, THI supplementation modulated energy metabolism and respiratory chain disturbance in LPS-induced RECs through suppression of the p38 MAPK pathway, which conformed to an increased ATP level. As a downstream metabolic regulator of AMPK, p38 is activated by transcription of AMPK to mediate the absorption of nutrients [59]. The overexpression of the p38 gene activates the MAPK pathway, which revealed the existence of potential interaction sites. Bewilderingly, the overexpression of the p38 gene did not affect the protein levels of SIRT1 and TLR4. Thus, it is uncertain whether THI directly modulates the inflammatory response and energy metabolism by suppression of the p38 pathway; however, that it involved p38 is clear.

## 5. Conclusions

**Figure 9:**
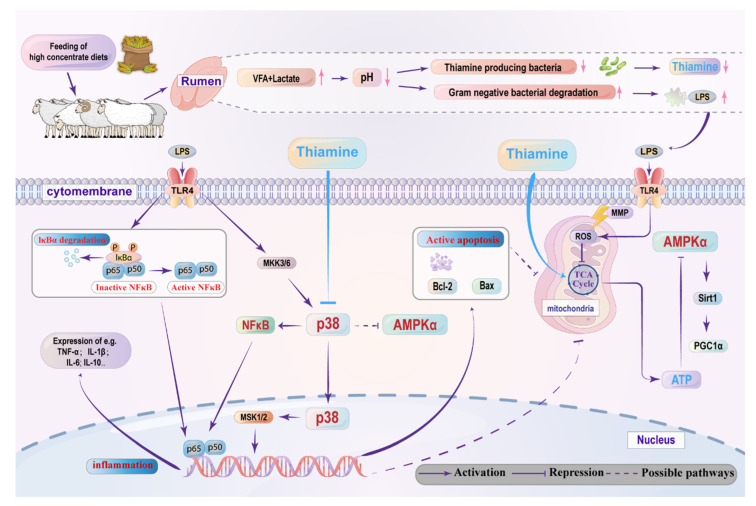
The potential mechanisms by which THI supplementation attenuates LPS-induced inflammation and energy metabolism disturbance in goats. The dietary change from forage to high concentrate (HC) diets causes low pH in the rumen, which thus results in LPS accumulation and thiamine deficiency. On the one hand, thiamine supplementation suppresses the ruminal epithelium inflammatory response by suppressing the p38 MAPK signaling pathway mediates inactivation of the NFκB factor. On the other hand, thiamine supplementation protects mitochondrial function, regulates activation of the AMPK pathway and thereby promotes ATP production, which may inhibit cell apoptosis. In addition, thiamine supplementation modulates energy metabolism disturbance and lipopolysaccharide-induced inflammation that may be related to the interaction effect between the AMPK and p38 MAPK signal pathway.

This study showed that THI supplementation has anti-inflammatory and energy metabolic-modulatory effects in RECs. These result largely from suppression of the NF-κB/p38 MAPK/AMPK signaling pathways. Additionally, we also revealed that THI supplementation can inhibit LPS-induced OD and apoptosis to protect mitochondrial function (Figure 9). Unfortunately, due to the complex molecular mechanisms, we could not fully determine whether THI regulates inflammation and energy metabolism via direct inhibition of p38, and potential targets of THI for regulation of inflammatory response and energy metabolism remain unidentified. In general, THI could serve as a potential supplement to relieve rumen inflammation and metabolic imbalances in ruminants.

## Data Availability

All data relevant to the study are included in the article or uploaded as supplementary information. Data are available on reasonable request. Data generated and analyzed during this study are available from the corresponding author on reasonable request.

## Supplementary Materials

The following supporting information can be downloaded at: https://www.mdpi.com/article/10.3390/antiox11102048/s1, Table S1: Primers for quantitative real-time PCR; Figure S1: Immunofluorescence identification of cytokeratin 18 in isolated goat ruminal epithelial cells.

## References

[1] Plaizier J., Danesh M., Derakhshani H., Golder H., Khafipour E., Kleen J., Lean I., Loor J., Penner G., Zebeli Q. (2018). Review: Enhancing gastrointestinal health in dairy cows. Animal.

[2] Owens F., Secrist D., Hill W., Gill D. (1998). Acidosis in cattle: A review. J. Anim. Sci..

[3] Nagaraja T., Titgemeyer E. (2007). Ruminal Acidosis in Beef Cattle: The Current Microbiological and Nutritional Outlook. J. Dairy Sci..

[4] Aschenbach J., Zebeli Q., Patra A., Greco G., Amasheh S., Penner G. (2019). Symposium review: The importance of the ruminal epithelial barrier for a healthy and productive cow. J. Dairy Sci..

[5] Khafipour E., Krause D., Plaizier J. (2009). A grain-based subacute ruminal acidosis challenge causes translocation of lipopolysaccharide and triggers inflammation. J. Dairy Sci..

[6] Zebeli Q., Ametaj B. (2009). Relationships between rumen lipopolysaccharide and mediators of inflammatory response with milk fat production and efficiency in dairy cows. J. Dairy Sci..

[7] Kent-Dennis C., Aschenbach J., Griebel P., Penner G. (2020). Effects of lipopolysaccharide exposure in primary bovine ruminal epithelial cells. J. Dairy Sci..

[8] Plaizier J., Khafipour E., Li S., Gozho G., Krause D. (2012). Subacute ruminal acidosis (sara), endotoxins and health consequences–sciencedirect. Anim. Feed. Sci. Tech..

[9] Ma Y., Elmhadi M., Zhang Y., Zhang H., Wang H. (2021). Thiamine Alleviates High-Concentrate-Diet-Induced Oxidative Stress, Apoptosis, and Protects the Rumen Epithelial Barrier Function in Goats. Front. Vet. Sci..

[10] Ma Y., Wang C., Elmhadi M., Zhang H., Han Y., Shen B., Wang H. (2021). Thiamine ameliorates metabolic disorders induced by a long-term high-concentrate diet and promotes rumen epithelial development in goats. J. Dairy Sci..

[11] Ma Y., Elmhadi M., Wang C., Zhang H., Wang H. (2021). Dietary supplementation of thiamine down-regulates the expression of mitophagy and endoplasmic reticulum stress-related genes in the rumen epithelium of goats during high-concentrate diet feeding. Ital. J. Anim. Sci..

[12] Kurashima Y., Goto Y., Kiyono H. (2013). Mucosal innate immune cells regulate both gut homeostasis and intestinal inflammation. Eur. J. Immunol..

[13] Ulivi V., Giannoni P., Gentili C., Cancedda R., Descalzi F. (2008). p38/NF-kB-dependent expression of COX-2 during differentiation and inflammatory response of chondrocytes. J. Cell. Biochem..

[14] Bonilla-Porras A., Jimenez-Del-Rio M., Velez-Pardo M. (2011). Vitamin K3 and vitamin Calone or in combination induced apoptosis in leukemia cells by a similar oxidative stress signalling mechanism. Cancer Cell Int..

[15] Zhang G., Zhang T., Jin Y., Liu J., Guo Y., Fan Y. (2018). Effect of caloric restriction and subsequent re-alimentation on oxidative stress in the liver of Hu sheep ram lambs. Anim. Feed. Sci. Technol..

[16] Nozaki S., Mizuma H., Tanaka M., Jin G., Tahara T., Mizuno K. (2009). Thiamine tetrahydrofurfuryl disulfide improves energy metabolism and physical performance during physical-fatigue loading in rats. Nutr. Res..

[17] Hernandez-Vazquez A., Garcia-Sanchez J., Moreno-Arriola E., Salvador-Adriano A., Ortega-Cuellar D., Velazquez-Arellano A. (2016). Thiamine deprivation produces a liver ATP deficit and metabolic and genomic effects in mice: Findings are parallel to those of biotin deficiency and have implications for energy disorders. Lifestyle. Genom..

[18] Dhir S., Tarasenko M., Napoli E., Giulivi C. (2019). Neurological, Psychiatric and Biochemical Aspects of Thiamine Deficiency in Children and Adults. Front. Psychiatry.

[19] Hardie D., Carling D., Carlson M. (1998). The AMP-activated/SNF1 protein kinase subfamily: Metabolic sensors of the eukaryotic cell. Annu. Rev. Biochem..

[20] Velazquez-Arellano A., Hernandez-Vazquez A. (2020). Vitamins as Cofactors for Energy Homeostasis and Their Genomic Control, With Special Reference to Biotin, Thiamine, and Pantothenic Acid. Principles of Nutrigenetics and Nutrigenomics.

[21] Zhao Y., Pan X., Zhao J., Wang Y., Peng Y., Zhong C. (2009). Decreased transketolase activity contributes to impaired hippocampal neurogenesis induced by thiamine deficiency. J. Neurochem..

[22] Pan X., Yang L., Beckers Y., Xue F., Tang Z., Jiang L., Xiong B. (2017). Thiamine supplementation facilitates thiamine transporter expression in the rumen epithelium and attenuates high-grain-induced inflammation in low-yielding dairy cows. J. Dairy Sci..

[23] Zhang H., Peng A., Zhao F., Yu L., Wang M., Osorio J., Wang H. (2020). Thiamine ameliorates inflammation of the ruminal epithelium of Saanen goats suffering from subacute ruminal acidosis. J. Dairy Sci..

[24] Brent B. (1976). Relationship of acidosis to other feedlot ailments. J. Anim. Sci..

[25] Gálfi P., Neogrady S., Kutas F. (1980). Culture of epithelial cells from bovine ruminal mucosa. Vet. Res. Commun..

[26] Gao J., Xu Q., Wang M., Ouyang J., Tian W., Feng D., Loor J. (2020). Ruminal epithelial cell proliferation and short-chain fatty acid transporters in vitro are associated with abundance of period circadian regulator 2 (PER2). J. Dairy. Sci..

[27] Liu J., Timm D., Hurley T. (2006). Pyrithiamine as a substrate for thiamine pyrophosphokinase. J. Biol. Chem..

[28] Graham C., Simmons N. (2005). Functional organization of the bovine rumen epithelium. Am. J. Physiol.-Reg. I..

[29] Zhang G., Deng Y., Zhang Y., Fan Y., Wan H., Nie Z., Wang F., Lei Z. (2016). Effect of PGC-1α over-expression or silencing on mitochondrial apoptosis of goat luteinized granulosa cells. J. Bioenerg. Biomembr..

[30] Zhang H., Zhao F., Peng A., Guo S., Wang M., Elsabagh M., Wang H. (2020). L-Arginine Inhibits Apoptosis of Ovine Intestinal Epithelial Cells through the l-Arginine–Nitric Oxide Pathway. J. Nutr..

[31] Xing X., Jiang X., Tang P., Wang Y., Li Y., Sun G., Zou S. (2016). Sodium butyrate protects against oxidative stress in HepG2 cells through modulating Nrf2 pathway and mitochondrial function. J. Physiol. Biochem..

[32] Gao D., Xu Z., Zhang X., Wang H., Wang Y., Min W. (2013). Molecular cloning, immunohistochemical localization, characterization and expression analysis of caspase-9 from the purse red common carp (*Cyprinus carpio*) exposed to cadmium. Aqua. Toxicol..

[33] Livak K., Schmittgen T. (2001). Analysis of relative gene expression data using real-time quantitative PCR and the 2−ΔΔCT method. Methods.

[34] Cheng J., Zhang Y., Ge Y., Li W., Cao Y., Qu Y., Liu J. (2020). Sodium butyrate promotes milk fat synthesis in bovine mammary epithelial cells via GPR41 and its downstream signalling pathways. Life Sci..

[35] Chang G., Zhuang S., Seyfert H., Zhang K., Xu T., Jin D., Guo J., Shen X. (2015). Hepatic TLR4 signaling is activated by LPS from digestive tract during SARA, and epigenetic mechanisms contribute to enforced TLR4 expression. Oncotarget.

[36] Manuel D., Madsen K., Churchill T., Dunn S., Ametaj B. (2007). Acidosis and lipopolysaccharide from Escherichia coli B:055 cause hyperpermeability of rumen and colon tissues. J. Dairy Sci..

[37] Pan X., Yang L., Xue F., Xin H., Jiang L., Xiong B., Beckers Y. (2016). Relationship between thiamine and subacute ruminal acidosis induced by a high-grain diet in dairy cows. J. Dairy Sci..

[38] Pan X., Xue F., Nan X., Tang Z., Wang K., Beckers Y., Xiong B. (2017). Illumina sequencing approach to characterize thiamine metabolism related bacteria and the impacts of thiamine supplementation on ruminal microbiota in dairy cows fed high-grain diets. Front. Microbiol..

[39] Zhu E., Fang L., Subramanian V., Said H., Sassoon C. (2015). Lipopolysaccharide and cytokines inhibit thiamine uptake and thiamine transporter gene expression in C2c12 myoblasts. Am. J. Respir. Crit. Care Med..

[40] Fan J., Feng G., Huang L., Tang G., Jiang H., Xu J. (2014). Naofen promotes TNF-alpha-mediated apoptosis of hepatocytes by activating caspase-3 in lipopolysaccharide-treated rats. World. J. Gastroenterol..

[41] Li J., Yuan J. (2008). Caspases in apoptosis and beyond. Oncogene.

[42] Campbell K., Rocha S., Perkins N. (2004). Active Repression of Antiapoptotic Gene Expression by RelA(p65) NF-kB. Mol. Cell..

[43] Lee J., Jung W., Jeong M., Yoon T., Kim H. (2012). Sanguinarine induces apoptosis of HT-29 human colon cancer cells via the regulation of Bax/Bcl-2 ratio and caspase-9-dependent pathway. Int. J. Toxicol..

[44] Zhang H., Ma Y., Wang M., Elsabagh M., Loor J., Wang H. (2020). Dietary supplementation of l-arginine and N-carbamylglutamate enhances duodenal barrier and mitochondrial functions and suppresses duodenal inflammation and mitophagy in suckling lambs suffering from intrauterine-growth-restriction. Food. Funct..

[45] Li X., Zhou A., Li X. (2007). Inhibition ofLycium barbarum polysaccharides andGanoderma lucidumpolysaccharides against oxidative injury induced by γ-irradiation in rat liver mitochondria. Carbohyd. Polym..

[46] Strandberg Y., Gray C., Vuocolo T., Donaldson L., Broadway M., Tellam R. (2005). Lipopolysaccharide and lipoteichoic acid induce different innate immune responses in bovine mammary epithelial cells. Cytokine.

[47] Lee S. (2015). Intestinal permeability regulation by tight junction: Implication on inflammatory bowel diseases. Intest. Res..

[48] Kowaltowski A., De-Souza-Pinto N., Castilho R., Vercesi A. (2009). Mitochondria and reactive oxygen species. Free Radic. Biol. Med..

[49] Cole-Ezea P., Swan D., Shanley D., Hesketh J. (2012). Glutathione peroxidase 4 has a major role in protecting mitochondria from oxidative damage and maintaining oxidative phosphorylation complexes in gut epithelial cells. Free Radic. Biol. Med..

[50] Amaral M., Ribeiro R., Vanzela E., Barbosa-Sampaio H. (2016). Reduced AMPK alpha2 protein expression restores glucose-induced insulin secretion in islets from calorie-restricted rats. Int. J. Exp. Pathol..

[51] Fernandez-Marcos P., Auwerx J. (2011). Regulation of PGC-1a, a nodal regulator of mito-chondrial biogenesis. Am. J. Clin. Nutr..

[52] Liang H., Ward W. (2006). PGC-1α: A key regulator of energy metabolism. Adv. Physiol. Educ..

[53] Hamano Y., Okada S., Tanaka T. (1999). Effects of thiamine and clenbuterol on body composition, plasma metabolites and hepatic oxygen consumption in broiler chicks. Br. Poult. Sci..

[54] Ye J., Zhu N., Sun R., Liao W., Fan S., Shi F., Ying Y. (2018). Metformin inhibits chemokine expression through the AMPK/NF-κB signaling pathway. J. Interf. Cytok. Res..

[55] Quivy V., Van Lint C. (2004). Regulation at multiple levels of NF-kappaB-mediated transactivation by protein acetylation. Biochem. Pharmacol..

[56] Saccani S., Pantano S., Natoli G. (2002). p38-Dependent marking of inflammatory genes for increased NF-κB recruitment. Nat. Immunol..

[57] Kefaloyianni E., Gaitanaki C., Beis I. (2006). ERK1/2 and p38-MAPK signalling pathways, through MSK1, are involved in NF-κB transactivation during oxidative stress in skeletal myoblasts. Cell. Signal..

[58] Chew J., Biswas S., Shreeram S., Humaidi M., Wong E., Dhillion M., Teo H., Hazra A., Fang C., Lopez-Collazo E. (2009). WIP1 phosphatase is a negative regulator of NF-κB signalling. Nat. Cell. Biol..

[59] Cheng Z., Pang T., Gu M., Gao A., Xie C., Li J. (2006). Berberine-stimulated glucose uptake in L6 myotubes involves both AMPK and p38 MAPK. BBA-GEN. Subjects.

