# Supplementary material for: Thiamine Supplementation Alleviates Lipopolysaccharide-Triggered Adaptive Inflammatory Response and Modulates Energy State via Suppression of NFκB/p38 MAPK/AMPK Signaling in Rumen Epithelial Cells of Goats
Source: Antioxidants (Basel). 2022 Oct 18;11(10):2048. doi: 10.3390/antiox11102048 (PMC9598694; doi:10.3390/antiox11102048)
Supplement: Supplementary file 1 [file antioxidants-11-02048-s001.zip › antioxidants-1951297-supplementary.pdf]

Supplementary Table.

Table S1. Primers for quantitative real-time PCR.

Gene Name[1]	Sequences (5′-3′)[2]	GenBank accession	Amplicon size	Efficiency[3] (%)
Inflammation				
IL-1β	**F:** CATGTGTGCTGAAGGCTCTC	XM_013967700.2	172	93.50
	**R:** AGTGTCGGCGTATCACCTTT			
IL-6	**F:** ACACTGACATGCTGGAGAAGATGC	NM_001285640.1	116	91.26
	**R:** CCGAATAGCTCTCAGGCTGAACTG			
IL-10	**F:** AAACAAGAGCAAGGCGGTGGAG	XM_005690416.3	83	95.82
	**R:** ACTCACTCATGGCTTTGTAGACACC			
TNF-α	**F:** CAAGTAACAAGCCGGTAGCC	XM_005696606.3	153	101.30
	**R:** AGATGAGGTAAAGCCCGTCA			
MAPK14	**F:** ACAGATGACCACGTTCAGTTCCTTATC	XM_018038791	130	103.55
	**R:** TCTTCAGCTCACAGTCTTCATTCACAG			
Thiamine transport				
SLC19A2	**F:** GAGGAACAGGAATCCAAGCCAGAC	XM_018060285.1	90	96.22
	**R:** GAGCAGAGGGCGAGAGGAGTAG			
SLC19A3	**F:** TGGACCTACTCTTACCTGGCACTAC	XM_005676643.3	84	97.88
	**R:** CTGGAGGATGATGACTGGCTTGTAG			
SLC25A19	**F:** CAGCTCCTTGAAGCGTGCCTAC	XM_013972239.2	126	92.15
	**R:** TCCAGTGGGTATGTGAGGGTCTTG			
Proliferation				
CCND1	**F:** TCTCCTATCACCGCCTGACA	XM_018043271.1	139	102.63
	**R:** TTGGGGTCCAAGTTCTGCTG			
CCNA2	**F:** TCCTTGAAGCTGACCCGTTC	XM_018056659.1	226	95.13
	**R:** TCCCTGATTGCTTGCTGAGG			

CDK2	**F:** AAGTGGCTGCATCACAAGGA	XM_013963900.1	150	90.86
	**R:** CTGGCCAAACCACCTCATCT			
CDK6	**F:** GACCAGCAGTATGAGTGCGT	XM_018047425.1	118	98.50
	**R:** TCTGCACTCTCACACGCTTC			
Energy metabolism				
AMPKα1	**F:** GAGATCAGGCTCAGTTAGCAACTATCG	XM_018065500.1	122	90.54
	**R:** CAGGAGAAGAGTCAAGTGAGGTTAAG			
AMPKα2	**F:** TGGTGGAGCAGAGGTCTGGTTC	XM_018044652.1	137	97.70
	**R:** GGTCAAGGAGCCCGTGAGAGAG			
SIRT1	**F:** TCTTGCGGCAGTAACAGTGATAGTG	XM_018042148.1	116	92.77
	**R:** ACATCAGCATCATCTTCCAAACCATTG			
PGC1α	**F:** GGCAGATACACTCTTCCACAGATTCC	NM_001285631.1	120	93.62
	**R:** CGGCTCGGATTTCCTGGTCTTG			
Respiratory chain				
Complex I	**F:** GAGCGGATTCACACCACAGACAG	XM_005680980.3	137	95.45
	**R:** TCAGAAGACAGCCAGCGGTAGG			
Complex II	**F:** CCAGGGAGGACTTCAAGGAGAGG	XM_018065656.1	87	103.20
	**R:** AGTGCTGCTCAAACGGCTTCTTC			
Complex III	**F:** GGCTCTAGCTGCTGGTTCATACAC	XM_005697593.3	130	92.85
	**R:** TCCACTTGCTGCCATTGACTTCC			
Complex IV	**F:** CTTTGCCTCTGAAGTCCTTGAGTCG	XM_005697738.3	150	94.30
	**R:** CCGAGTGGAGCCAGGAGTTGAG			
Complex V	**F:** GACTGCCTCCCATCCTAAATGCC	XM_005680331.2	125	105.48
	**R:** CAAGCCTTCTGTACCGTCCATAGC			
Reference genes				
β-actin	**F:** GAATCCTGCGGCATTCACG	NM_001314342.1	189	95.90
	**R:** GGGCGCGATGATCTTGA			
GAPDH	**F:** GGGTCATCATCTCTGCACCT	XM_005680968.3	176	101.76

	**R:** GGTCATAAGTCCCTCCACGA			
HPRT1	**F:** CACCAGCTGGCTCCGTTATG	XM_018044253.1	163	95.04
	**R:** AGTCGTTCGGTCCTGTCCAT			

[1]*IL-1β* = interleukin-1β; *TNF-α* = tumor necrosis factor alpha; *MAPK14* = mitogen-activated protein kinase 14 (p38); *SLC25A2* = solute carrier family 19, member 2 (THTR1); *SLC19A3* = solute carrier family 19, member 3 (THTR2); *SLC25A19* = mitochondrial thiamine pyrophosphate transporter (MTPPT); *CCND1* = cyclin D1; *CCNA2* = cyclin A2; *CDK2* = cyclin dependent kinase 2; *AMPKα1* = protein kinase AMP-activated catalytic subunit alpha 1; *SIRT1* = sirtuin 1; *PGC1α* = PPARG coactivator 1 alpha; *GAPDH* = glyceraldehyde-3-phosphate dehydrogenase; *HPRT1* = hypoxanthine phosphoribosyltransferase 1.

[2]F = forward; R = reverse.

[3]Efficiency = $-1 + 10^{(-1/\mathrm{slope})} \times 100$.

Supplementary Figure.

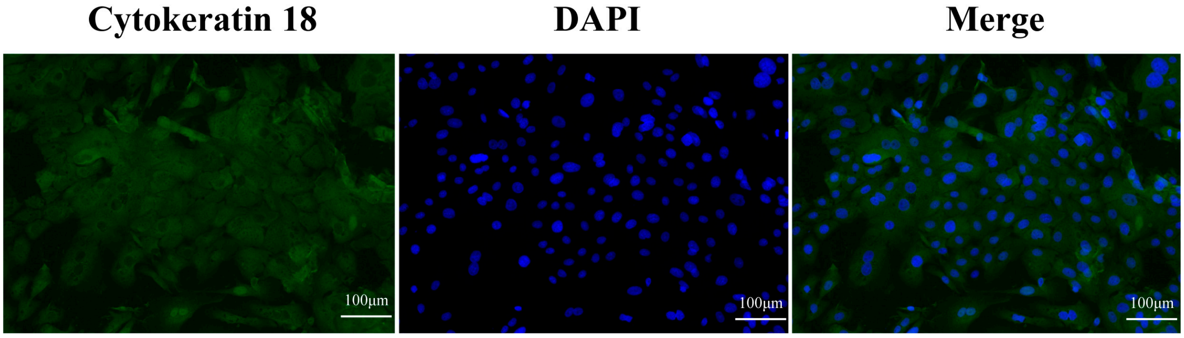

Figure S1. Immunofluorescence identification of cytokeratin 18 in isolated goat ruminal epithelial cells. The primary antibody was Cytokeratin 18 (Santa Cruz, sc-32329). Green fluorescence denotes stained proteins and blue denotes the cell nucleus stained with DAPI solution
